# Identification and molecular characterization of mutant line deficiency in three waxy proteins of common wheat (*Triticum aestivum* L.)

**DOI:** 10.1038/s41598-021-82865-2

**Published:** 2021-02-10

**Authors:** Qian Liu, Yaping Hu, Mengyun Hu, Lijing Sun, Xiyong Chen, Qianying Li, Peinan Wang, Li-an Wang, Yingjun Zhang, Hui Li

**Affiliations:** 1 Institute of Cereal and Oil Crops, Hebei Academy of Agriculture and Forestry Sciences, Hebei Laboratory of Crop Genetics and Breeding, 162 Hengshan Street, Shijiazhuang, 050035 China; 2 Xingtai Medical College, 618 Gangtie North Road, Xingtai, 054000 China; 3 College of Life Sciences, Hebei Normal University, 20 Road East. 2nd Ring South, Shijiazhuang, 050024 China

**Keywords:** Genetics, Molecular biology, Plant sciences

## Abstract

Starch is the main component of wheat (*Triticum aestivum* L.) grain and a key factor in determining wheat processing quality. The *Wx* gene is the gene responsible for amylose synthesis. An ethyl methanesulfonate (EMS) mutagenized population was generated using common wheat cv. Gao 8901, a popular and high-quality cultivar in China. A waxy mutant (Wx-null) was isolated by screening M3 seeds with KI-I_2_ staining of endosperm starch. No obvious waxy proteins in Wx-null line were detected using Sodium dodecyl sulfate-polyacrylamide gel electrophoresis (SDS-PAGE). DNA sequencing revealed three SNPs and a 3-bp InDel in the first exon, and a 16-bp InDel at the junction region of the first *Wx-A1* intron from the Wx-null line. Six SNPs were identified in *Wx-B1* gene of Wx-null line compared to the wild-type Gao 8901, including four missense mutations. One nonsense mutation was found at position 857 in the fourth exon, which resulted in a premature stop codon. Expression levels of *Wx* genes were dramatically reduced in the Wx-null line. There were no detectable differences in granule size and morphology between Wx-null and wild-type, but the Wx-null line contained more B-type starch granules. The amylose content of the Wx-null line (0.22%) was remarkably lower compared to the wild-type Gao 8901 (24.71%). Total starch is also lower in the Wx-null line. The Wx-null line may provide a potential waxy material with high agronomic performance in wheat breeding programs.

## Introduction

Wheat (*Triticum aestivum* L.) is one of the most important crops worldwide. Starch is the main component of wheat grain and accounts for 60–75% of seed dry mass. Wheat starch consists of two molecular types of glucose polymers: amylose and amylopectin. Based on the content of amylose and amylopectin in starch, wheat is classified into non-waxy wheat starch, usually consisting of 18–29% amylose^1^, and waxy wheat starch, consisting mostly of amylopectin and less than 2% of amylose^2^.

It is generally known that in the endosperm, amylose is primarily synthesized by GBSS I (the ‘waxy’ protein)^3^. Wild-type wheat carries three functional *Wx* genes (*Wx-A1a*, *Wx-B1a*, and *Wx-D1a*) on chromosomes 7AS, 4AL (translocated from the original 7BS) and 7DS, respectively^4,5^. Amylose content in wheat starch is affected by GBSS I activity^6^. When one or two *Wx* genes are non-functional, wheat often produces starch with reduced amylose content and is known as ‘partial waxy’^7^. If all three *Wx* genes are null or non-functional, grains almost entirely consist of amylopectin and are termed ‘waxy’^3,4,8^. *Wx* genes have additive but unequal effects on amylose content; *Wx-B1* has the greatest effect on the amylose synthesis, followed by *Wx-D1* and *Wx-A1*^6,9^.

Starch is a key factor determining the processing quality of wheat^10^. Amylose and amylopectin differ significantly in their physicochemical properties. Amylose is primarily responsible for gel formation, especially regarding its structure and crystallinity^11^, while amylopectin has an important role in retrogradation and long-term rheological characteristics of starch gels^12^. As a result, changes in amylose or amylopectin content have profound impacts on end-use quality of wheat^13^. Compared with wild-type wheat starch, waxy wheat starch generally has higher peak viscosity and swelling volume, which can improve the quality of noodles including Japanese udon, Chinese-style ra-men and yellow alkaline noodles^14–16^. Waxy starch can also influence dough mixing characteristics, bread-making quality and bread shelf-life^17^.

The development and selection of waxy wheat are of importance in wheat breeding programs, but natural waxy wheat does not occur in nature. Identification of waxy protein-deficient lines play an important role in developing waxy wheat. Firstly, two cultivars with lower amylose content, Kanto l07 (K107) and K79, were found among Japanese wheat cultivars and shown to be *Wx-A1*/*Wx-B1* double null lines^7,18^. Later a worldwide sample screening of 1960 wheat cultivars was performed, and one Chinese wheat (BaiHuo) was identified as null at the *Wx-D1* locus^5^. By crossing K107 and BaiHuo, Nakamura^8^ succeeded in producing the world’s first completely waxy hexaploid wheat that lacked all isoforms of GBSS I. Since then, searches for novel *Wx* alleles in common wheat continued, with the goal of finding genetic variability to enable breeders to develop wheat lines with diverse starch properties and end-use quality characteristics. Ninteen null alleles were found in *Wx-A1*, 17 alleles in *Wx-B1*, and 7 alleles in *Wx-D1*, respectively^10,19–29^. However, no cultivars lacking both Wx-A1 and Wx-D1 proteins, both Wx-B1 and Wx-D1 proteins, or all three Wx proteins had been detected^5,30^. Mutagenesis is a popular and effective way to create genetic variations in crops. Many *Wx* gene mutants have been isolated using EMS-induced assays^29,31–34^. Slade and colleagues^35^ first time produced a wx-null genotype using a TILLING approach in bread wheat. Later Botticella^36^ reported another TILLING wx-null genotype. In the present study, by screening about 9000 seeds from a mutant population of bread wheat cultivar Gao 8901 treated with EMS solution we were lucky to obtain a triple mutant of *Wx* gene. The *Wx* null line provides breeders valuable germplasms suitable for the production of bread and high-quality salted noodles. It may also be used to provide one or more waxy null alleles with high agronomic performance in wheat breeding programs.

## Results

### Identification of the waxy protein null mutant line

Seeds of common wheat cv. Gao 8901 were treated with a 1.0% EMS solution, with a survival rate of M1 plants of 50–60%. M1 plants were self-fertilized to produce the M2 generation. Half-seed staining using iodine potassium iodine solution (0.1% I_2_/1% KI) was employed to screen for waxy protein mutants. About 9000 seeds from the M3 generation were screened. One seed showed brown–red color stained with I_2_-KI solution while others were dark blue (Supplementary Fig. S1). We hypothesized that this seed was a waxy protein-deficient mutant and named it Wx-null.

As the chance of acquiring a triple mutant is really low, it was important to make sure it was not a contaminating seed. The wild-type (Gao 8901) has obvious pubescence on its glume, which can easily distinguish it from other cultivars. Morphologically, the Wx-null line has the same characteristics as the wild type, such as plant height, plant shape, leaf shape, leaf color, spike shape, etc. Seven pairs of SSR primers were used to detect the mutant line and wild-type; they were *Xbarc80* (1BL), *Xgwm294* (2AL), *Xgdm72* (3DL), *Xbarc91* (4DL), *Xgwm67* (5BS), *Xgwm334* (6AS) and *Xgwm333* (7BL). The mutant line displayed the same band patterns as the wild type (Supplementary Fig. S2). So, it was determined that the mutant line was from the wild-type Gao 8901.

### Waxy protein pattern analysis by SDS-PAGE electrophoresis

The waxy protein pattern of the Wx-null line (M5 generation) was analyzed by SDS-PAGE electrophoresis. The wild-type Gao 8901 and two Wx-A1 null lines were used as controls. The molecular weight of three GBSS I isotypes (Wx-A1, Wx-B1, and Wx-D1 proteins) are about 62.8, 58.7 and 56.7 kD, respectively. Because the molecular weight difference between Wx-B1 and Wx-D1 proteins is small, the two bands are very close. The wild-type Gao 8901 showed three waxy protein bands, otherwise, no obvious bands were seen in the Wx-null mutant line (Supplementary Fig. S3).

### Amylose content and GBSS I activity decreased significantly in Wx-null line

**Figure 1:**
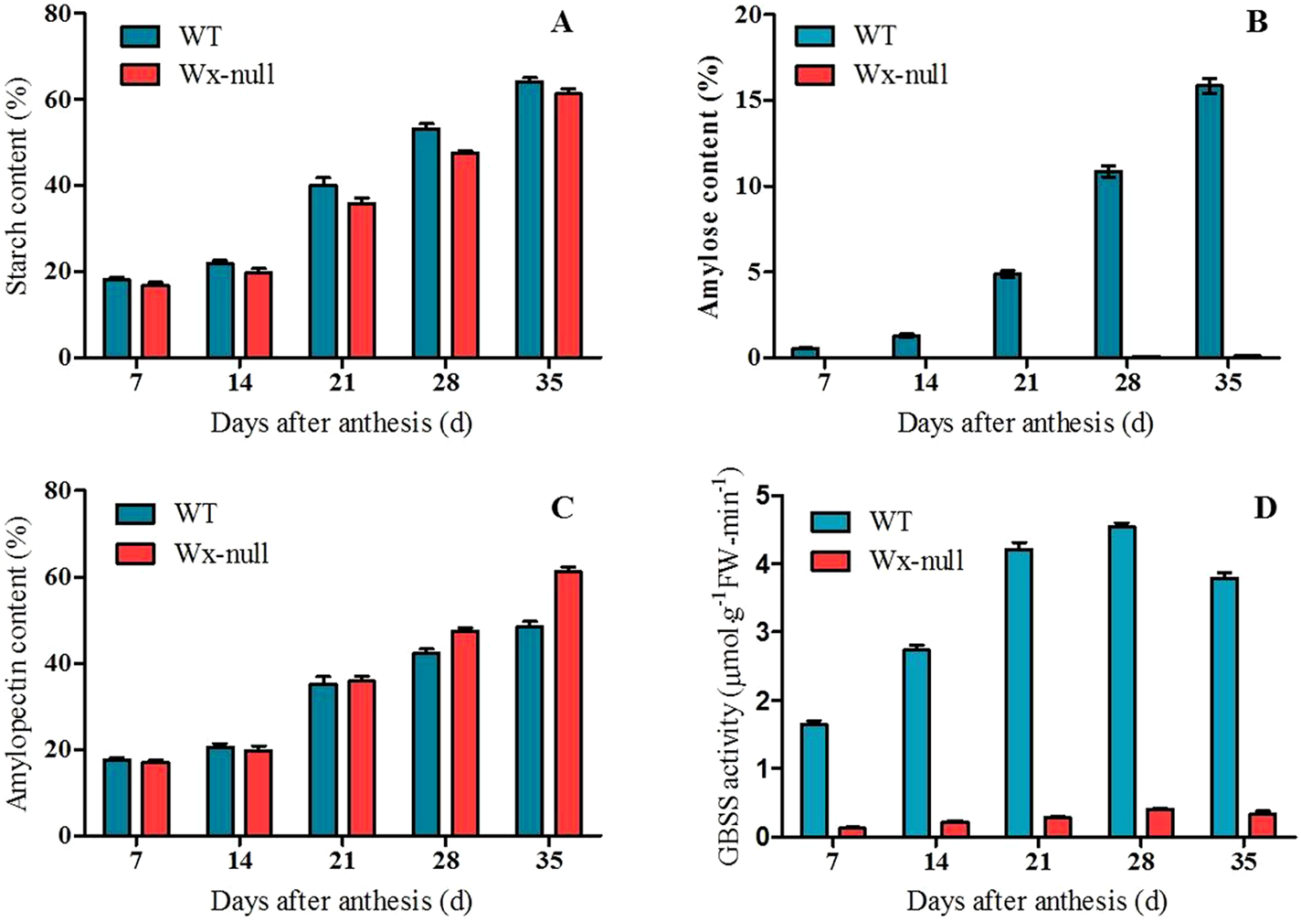
Total starch content, amylose content and GBSS I activity analysis. (**A**) Total starch content; (**B**) amylose content; (**C**) amylopectin content; (**D**) GBSS I activity. Error bars show SEM.

Total starch, amylose content and GBSS I activity were determined for wild-type Gao 8901 and mutant line Wx-null (Fig. 1). Total starch content in both wild-type and the Wx-null line increased continuously from 7 to 35 DAF, but the final starch content in Wx-null (61.4%) was lower than in the wild-type (64.3%) (*P* < 0.05). The amylose content in the Wx-null line (0.22%) was also significantly lower than in the wild-type (24.7%) (*P* < 0.01). The GBSS I activity reached the maximum value (4.54 ± 0.05) at 28 DAF, then decreased lightly. Weak GBSS I activity can be detected in the Wx-null line, showing a similar trend as wild-type; However, the activity at 28 DAF was only 0.41 ± 0.02.

### Starch granule characteristics of the Wx-null line

**Figure 2:**
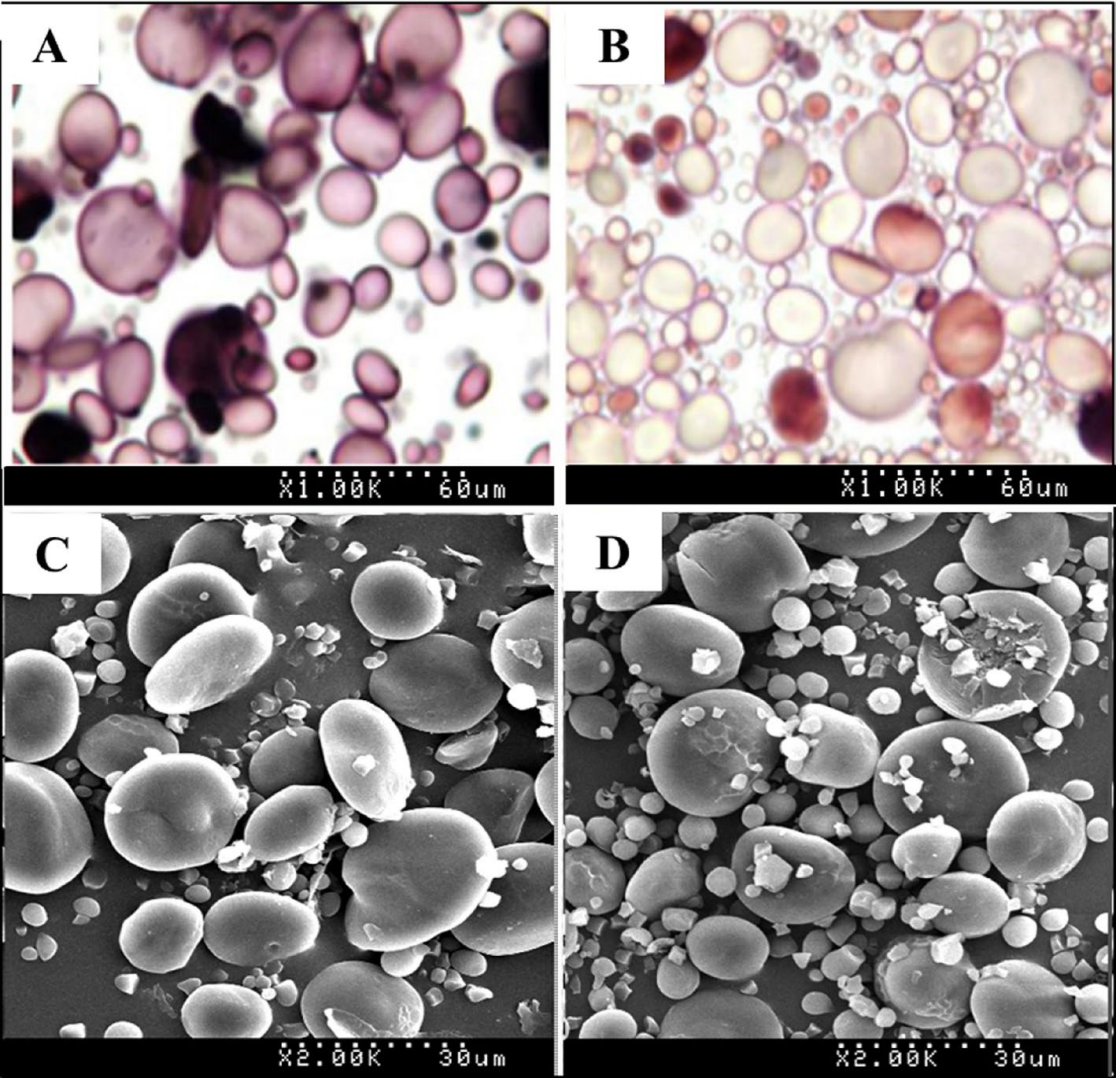
Starch granule staining and structure analysis. Starch granule staining by I_2_-KI solution (1000 diameters). (**A**) Wild-type; (**B**) Wx-null line. Starch structure visualized by SEM (2000 diameters). (**C**) wild-type; (**D**) Wx-null line. The total length of the white dots at the bottom of the picture represents 60 µm (1000 diameters) and 30 µm (2000 diameters), respectively.

Starch granule staining and scanning electron microscopy were employed to assess the effect of waxy protein deficiency on Wx-null line starch granule characteristics. When stained with Lugol, starch granules of wild-type Gao 8901 were dyed blue (Fig. 2A), while Wx-null line granules were stained red-brown (Fig. 2B). SEM observation of wild-type and Wx-null starch shapes revealed similar granule morphological features and size. A-type starch granules were over 10 μm in diameter and lenticular in shape, and B-type starch granules were less than 10 μm in diameter and roughly spherical in both the wild-type and the Wx-null line (Fig. 2C,D). The numbers of A-type and B-type starch granules in 5 fields of microscope view were counted, showing that the ratio of B-type/A-type of Wx-null (12.41 ± 3.56) was much higher than wild-type (5.19 ± 2.42).

### *Wx* gene sequences analysis of the Wx-null line

**Figure 3:**
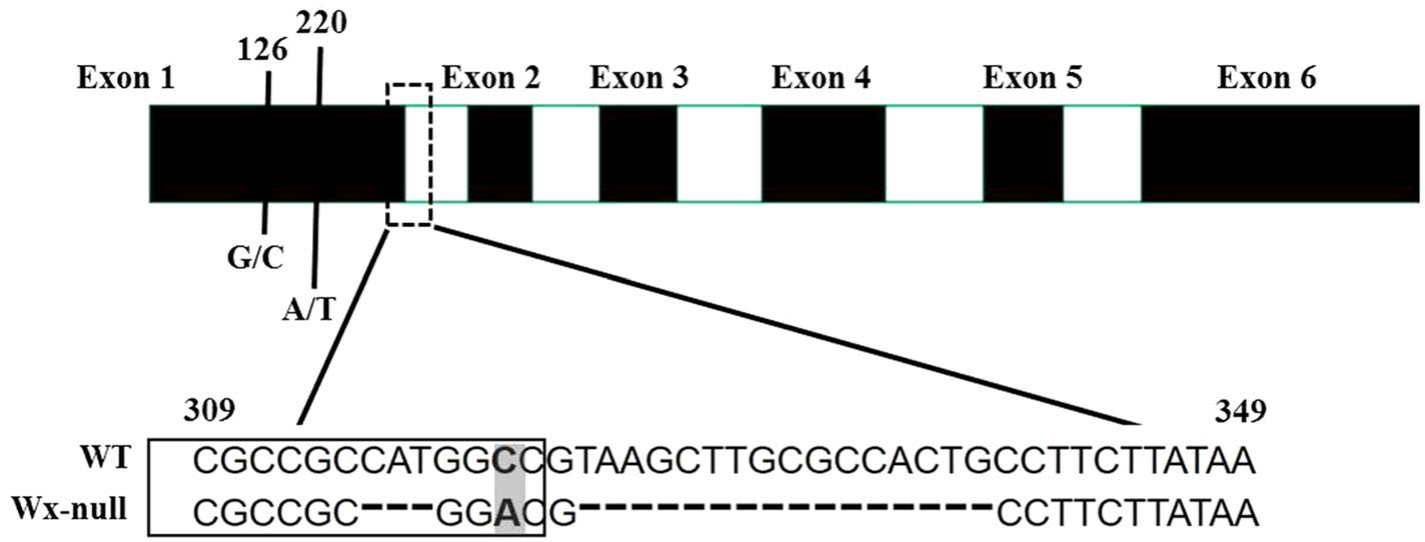
Analysis of *Wx-A1* gene sequences in wild-type and Wx-null line. Black and white boxes respectively indicate exons and introns. Numbers show base position. DNA sequences at the border of the first exon are boxed. SNP is indicated by the shaded area. Dashed line indicates the InDel.

**Figure 4:**
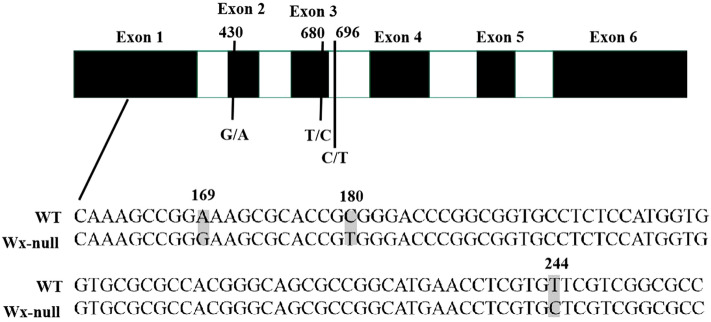
Analysis of *Wx-B1* gene sequences in wild-type and Wx-null line. Black and white boxes respectively indicate exons and introns. Numbers show base position. SNPs is indicated by the shaded area.

**Figure 5:**
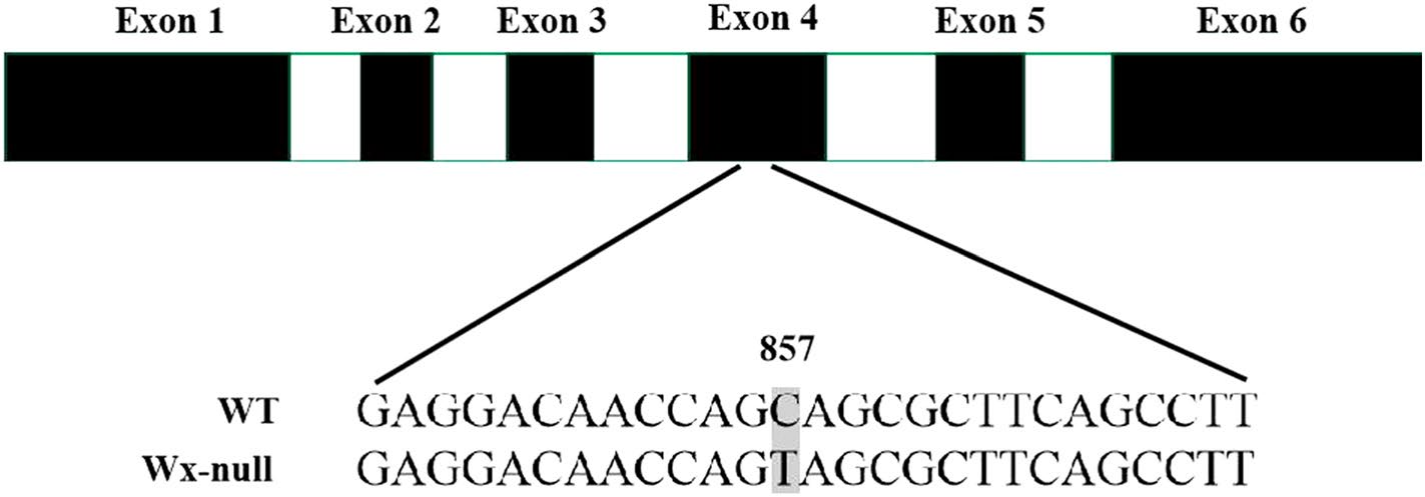
Analysis of *Wx-D1* gene sequences in wild-type and Wx-null line. Black and white boxes respectively indicate exons and introns. Numbers show base position. SNP is indicated by the shaded area.

Genomic DNA sequences of *Wx* genes of Gao 8901 were obtained. The gDNA of the *Wx* gene from genomes A, B and D were respectively 2781 bp, 2794 bp and 2862 bp in total. They were named *Wx-A1* (GenBank accession EU719608), *Wx-B1* (GenBank accession EU719610) and *Wx-D1* (GenBank accession EU719612), respectively. All *Wx* genes contained 11 exons and 10 introns. The gDNA sequences of *Wx* genes of the Wx-null line were also analyzed to detect mutant sites. DNA sequencing of the *Wx-A1* gene from the Wx-null line revealed three SNPs and a 3-bp insertions and deletions (InDel) in the first exon. The G/C mutant at position 126 and A/T mutant at position 220 resulted in amino acid changes from methionine to isoleucine and serine to cystine, respectively. The 3-bp InDel (CAT) and the third SNP occurred at positions 315–317 and 320, respectively. Furthermore, a 16-bp InDel (GGCCGTAAGCTTGCGCCAC) was found at the junction region in the first intron (Fig. 3). The Wx-null *Wx-A1* gene was named *Wx-A1-null* (GenBank accession EU719609). There was a total of six SNPs in the *Wx-B1* gene from the Wx-null line compared to wild-type Gao 8901, three of which were present in the first exon, one in the second, one in the third, and the last in the third intron (Fig. 4). The SNPs at positions 169 (A/G), 244 (T/C), 430 (G/A) and 680 (T/C) were missense mutations. The SNP (C/T) at position 180 was a same-sense mutation. The *Wx-B1* gene in the Wx-null line was named *Wx-B1-null* (GenBank accession EU719611). One nonsense mutation (CAG to TAG) was found at position 857 in the fourth exon in Wx-null *Wx-D1* gene line, which resulted in a premature stop codon (Fig. 5). The Wx-null *Wx-D1* gene was then named *Wx-D1-null* (GenBank accession EU719613).

### Prediction and comparison of secondary structures of Wx-B1 protein

**Figure 6:**
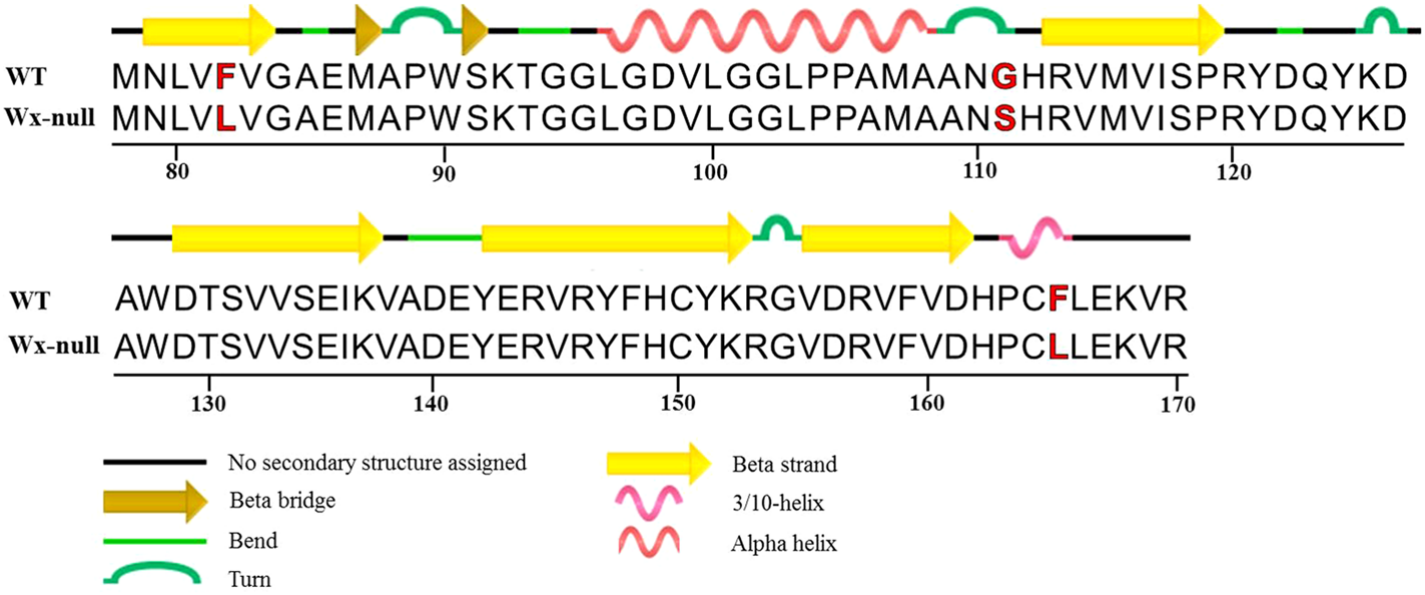
Protein sequence alignment and structural analysis. Numbers show residue position. Amino acid polymorphism is indicated by the red letter.

The four missense mutations in the *Wx-B1* at positions 169 (A/G), 244 (T/C), 430 (G/A) and 680 (T/C) resulted in amino acid changes from lysine (K) to glutamic acid (E), phenylalanine (F) to leucine (L), glycine (G) to serine (S), and phenylalanine (F) to leucine (L), respectively. Sequence alignments with rice GBSS I and structural analysis (PDB: 3VUF)^37^ were carried out. Phenylalanine (F) to leucine (L) at residue 82 just located at a beta strand, residue 111 (G–S) at a turn, and residue 165 (F–L) at a helix region, respectively (Fig. 6).

### Expression level of *Wx* gene decreases significantly in the Wx-null line

RT-PCR was carried out to determine *Wx* gene expression level changes between wild-type Gao 8901 and the Wx-null line. Total RNA was isolated from the seeds of Wx-null and wild-type at 15 DAF, reverse-transcribed into cDNA and amplified by PCR. The *Actin* gene had the same PCR amplification level in both samples, indicating that their cDNA was at equal concentrations. The forward and reverse RT-PCR primers for *Wx* gene located in the exon 3–exon 4 junction region and in exon 6, respectively. As the sequence identity of Wx-A1, Wx-B1 and Wx-D1 is very high (more than 95%), the RT-PCR primers were not gene specific. The amplified fragments of the Wx-A1, Wx-B1 and Wx-D1 were the same length (568 bp) showing one band in the gel. The *Wx* genes were strongly expressed in seeds of wild-type Gao 8901. However, expression levels of *Wx* genes was significantly decreased in the Wx-null line (Supplementary Fig. S4).

## Discussion

Pre-mRNA alternative splicing (AS) plays an important role in gene expression diversity in eukaryotes. It is also estimated that a significant fraction of genes (about 20%) undergo AS in plants^38,39^. InDel contributes to the generation of diversity in AS isoforms^40,41^, by affecting splicing efficiency^42^, the stability of the pre-mRNA structure, or the expression level of correctly spliced transcripts^43^. Through DNA sequencing, we identified three SNPs and a 3-bp InDel in the first exon, and a 16-bp InDel at the junction region in the first intron in the *Wx-A1* gene of the Wx-null line (Fig. 3). The 16-bp InDel destroyed the GT-AT intron–exon boundary, which may have induced AS and loss of function of the *Wx-A1* gene. The first reported null allele *Wx-A1b* (from K107) had a 23 bp deletion (in the second intron) also located at the exon–intron junction^44^. Additionally, Luo et al.^29^ reported an SNP (G–A) at the splicing site within the eighth intron, which caused incorrect RNA splicing and gene inactivation, suggesting a similar molecular mechanism for the above null *Wx-A1* alleles.

*Wx-D1* gene has the lowest variant frequency, and null *Wx-D1* alleles are extremely rare in common wheat^45^. Previously, researchers described a 588-bp fragment deletion in *Wx-D1b*^44^, a 724-bp fragment deletion in *Wx-D1h*^46^ and a nucleotide substitution in *Wx-D1g*^47^ among others. In this study, molecular characterization of *Wx-D1* gene of the Wx-null line showed one nonsense mutation (from CAG to TAG) in the fourth exon resulting in a premature stop and non-function waxy protein. Nonsense mutations on the allele *Wx-D1* had also been reported in previous studies^35,48,49^.

The total starch content decreased in the Wx-null line (61.4%) compared to wild-type Gao 8901 (64.3%), probably because GBSS I enzyme deficiency causes a significant reduction in total starch biosynthesis though it does not affect amylopectin synthesis^50^. The amylose content was very low in the Wx-null line (0.22%), but it was not zero (Fig. 1). Furthermore, a faint band of *Wx* gene expression was observed in the Wx-null line in RT-PCR figure (Supplementary Fig. S4). Then, GBSS I activity analysis was performed to confirm the results. It can be seen that weak GBSS I activity can be detected in Wx-null line (Fig. 1). As the *Wx-A1* and *Wx-D1* were frameshift and nonsense mutations, respectively, the reason may be that the amino acid changes in Wx-B1 protein may dramatically reduce its activity, but can not cause complete loss of its function.

## Methods

### Plant materials

The Chinese common wheat cv. Gao 8901 was selected for developing EMS treated mutant lines. Gao 8901 has good baking-quality and is widely planted in the Huang and Huai winter wheat region. Approximately 5000 dry seeds of Gao 8901 were soaked in distilled water for 12 h before being treated with 1.0% EMS at room temperature (25–27 °C) for 6 h and then washed under running tap water for 4 h prior to seeding. In the subsequent growing season, treated seeds (M0) were individually sown and self-fertilized to produce the M1 generation. All the M1 plants were harvested and sown to produce the M2 generation. One spike per M2 plant was picked, mixed and threshed. The seeds (M3) were used for screening waxy protein mutants. Young leaves of *Wx* mutants were collected for DNA extraction and seeds were planted in a plant-to-row fashion to produce the M5 generation. The M5 seeds were used for RNA extraction, protein electrophoresis, amylose content determination, starch granule staining, and scanning electron microscopy analysis. All the field trials were conducted at the experimental station in Shijiazhuang, Hebei province.

### Half-seed screening of *Wx* mutant lines

Waxy and non-waxy wheat seeds can be easily distinguished by staining. Starch from non-waxy wild-type containing amylose generates blue-black complexes with iodine, while starch from waxy protein mutants without amylose stains red-brown. M3 seeds were cut horizontally in two parts containing embryo and endosperm. The non-embryo half part of seed was dipped into an iodine potassium iodine solution (0.1% I_2_/1% KI) for 1 min. The seeds dyed red-brown were selected, and the embryo portion of the seed were used to grow to a plant.

### Electrophoresis of waxy proteins

Waxy proteins were extracted following published methods^51^ and separated using sodium dodecyl sulfate–polyacrylamide gel electrophoresis (SDS-PAGE) in a discontinuous buffer system (pH: 6.8/7.8). The polyacrylamide concentrations of stacking gel and resolving gel were respectively 4.5% and 15%. Electrophoresis was performed at a constant voltage of 180 V for stacking gel and 220 V for resolving gel. Run time was approximately 4 h just after the tracking dye (bromophenol blue) had migrated off the gel. After electrophoresis, waxy proteins were detected by silver staining.

### Sequence analysis of *Wx* genes

Genomic DNA was extracted from young leaves of *Wx* mutants (M4). Because the genomic sequences of *Wx* genes are about 2.8 kb and have high similarity at the 5′ and 3′ ends among A, B and D genomes, overlapping PCR was employed to amplify the complete *Wx* sequences. Primers were designed based on alignments of *Wx* genes sequences from the NCBI database (GenBank accession AB019622, AB019623, and AB019624) and primers reported by Slade and colleagues^35^. Primers were synthesized by Shanghai Sangon Biotech Co., Ltd. (http://www.sangon.com) and described in Supplementary Table S1. PCR was performed in volumes of 20 μl, including 2 μl of 10 × Ex Taq buffer, 0.5 μl of dNTP (2.5 mM of each dNTP), 1 μl of each primer (5 μM), 1 U of ExTaq and 80 ng of template DNA. All reagents were from Takara Biotechnology Co., Ltd. (http://www.takara.com.cn). Reaction conditions were 94 °C for 5 min, followed by 8 cycles at 94 °C for 30 s, annealing at 70–65 °C for 30 s (− 1 °C per cycle), 72 °C for 1 min, then 30 cycles at 94 °C for 30 s, annealing at 57–63 °C for 30 s (depending on the primer), 72 °C for 1 min and a final extension at 72 °C for 10 min. PCR products were separated by electrophoresis in 1% agarose gels stained with ethidium bromide and visualized using UV light. Targeted bands were recovered and cloned into the pEASY-T1 simple vector (Beijing TransGen Biotech Co., Ltd. http://www.transgen.com.cn) and sequenced by Shanghai Sangon Biotech Co., Ltd (http://www.sangon.com). Sequence analysis and characterization were performed using the software DNAMAN (http://www.lynnon.com) and Vector NTI Advance 10 (http://www.invitrogen.com). To guarantee sequence accuracy, PCR and DNA sequencing was repeated.

### Expression level analysis of *Wx* genes in mutant line using RT-PCR

Total RNA was isolated from seeds of both *Wx* mutant line and wild-type Gao 8901 with three biological replicates at 15 days after flowering (DAF) using the Trizol method (www.tiangen.com). All samples were DNase-treated before reverse transcription. The first-strand cDNA was synthesized by MMLV reverse transcriptase (http://www.promega.com) using oligo (dT) as a primer. Reverse transcriptional products were adjusted to an equal concentration according to the PCR signal generated from the internal standard *Actin* house-keeping gene and used as templates for RT-PCR. All primers used in RT-PCR are listed in Supplementary Table S1. RT-PCR was performed in total volumes of 20 µl, including 2 µl of 10 × ExTaq buffer, 0.5 µl of dNTP (2.5 mM of each dNTP), 1 µl of each primer (5 µM), 1U of ExTaq DNA polymerase and 80 ng of template cDNA. Reaction condition was denaturation at 94 °C for 3 min, followed by 20 cycles of 94 °C for 30 s, 59 °C for 30 s and 72 °C for 1 min, and a final extension of 72 °C for 5 min. RT-PCR products were separated in 1% agarose gels, and bands were visualized with ethidium bromide.

### Amylose content and GBSS I activity analysis

The seeds of 7, 14, 21, 28 and 35 DAF of mutant line and wild-type Gao 8901 were used to carry out starch content, amylose content and GBSS I activity analysis. Total starch and amylose contents were measured using the Total Starch Assay Kit and Amylose/Amylopectin Assay Kit (www.megazyme.com) according to manual instructions. GBSS I activity was measured using the Micro Bound Station amylosynthease Assay Kit (http://www.shkxbio.com). All tests were performed on three replicates. Student’s t-test assessed statistical differences between wild type and mutant line.

### Starch granule staining

A small amount of starch granules were put onto a glass slide with a toothpick followed by a drop of dye solution [50% (v/v) glycerol, 0.1% I_2_/1% KI (w/v)], then lightly covered with a cover slide and observed under a microscope.

### Scanning electron microscopy analysis

The granular morphology of starch was examined by scanning electron microscopy (SEM). Wheat seeds were transversely cut with a knife and fixed on circular aluminum stubs with double-sided sticky tape. Samples were sputter-coated with gold particles using an ionic sprayer (Eiko E-1020, Hitachi) and observed under a scanning electron microscope (S-570, Hitachi) with an accelerating voltage of 20 kV.

## Supplementary Information

Supplementary Information.

## Data Availability

All data generated or analyzed during this study are included in this published article and its Supplementary data files.

## References

[1] Singh S (2009). Relationship of granule size distribution and amylopectin structure with pasting, thermal, and retrogradation properties in wheat starch. J. Agric. Food Chem..

[2] Yasui T, Sasaki T, Matsuki J (2002). Starch properties of a bread wheat (*Triticum aestivum* L.) mutant with an altered flour-pasting profile. J. Cereal Sci..

[3] Graybosch RA (1998). Waxy wheats: Origin, properties, and prospects. Trends Food Sci. Technol..

[4] Nakamura T (1993). Identification of three Wx proteins in wheat (*Triticum aestivum* L.). Biochem. Genet..

[5] Yamamori M (1994). Waxy protein deficiency and chromosomal location of coding genes in common wheat. Theor. Appl. Genet..

[6] Saito M (2009). A novel codominant marker for selection of the null *Wx-B1* allele in wheat breeding programs. Mol. Breed..

[7] Nakamura T (1993). Decrease of waxy (Wx) protein in two common wheat cultivars with low amylose content. Plant Breed..

[8] Nakamura T (1995). Production of waxy (amylose-free) wheats. Mol. Gen. Genet..

[9] Miura H, Sugawara A (1996). Dosage effects of the three *Wx* genes on amylose synthesis in wheat endosperm. Theor. Appl. Genet..

[10] Yamamori M, Quynh N (2000). Differential effects of Wx-A1,-B1 and-D1 protein deficiencies on apparent amylose content and starch pasting properties in common wheat. Theor. Appl. Genet..

[11] Goodfellow B, Wilson R (1990). A Fourier transform IR study of the gelation of amylose and amylopectin. Biopolymers.

[12] Gudmundsson M (1994). Retrogradation of starch and the role of its components. Thermochim. Acta.

[13] Klucinec JD, Thompson DB (2002). Amylopectin nature and amylose-to-amylopectin ratio as influences on the behavior of gels of dispersed starch. Cereal Chem..

[14] Noda T (2001). Relationship between physicochemical properties of starches and white salted noodle quality in Japanese wheat flours. Cereal Chem..

[15] Baik BK (2003). Characteristics of noodles and bread prepared from double-null partial waxy wheat. Cereal Chem..

[16] Guo G (2003). Asian salted noodle quality: Impact of amylose content adjustments using waxy wheat flour. Cereal Chem..

[17] Bhattacharya M (2002). Staling of bread as affected by waxy wheat flour blends. Cereal Chem..

[18] Kuroda A (1989). A method of measuring amylose content and its variation in Japanese wheat cultivars and Kanto breeding lines. Jpn. J. Breed..

[19] Yamamori M, Guzmán C (2013). SNPs and an insertion sequence in five *Wx-A1* alleles as factors for variant Wx-A1 protein in wheat. Euphytica.

[20] Ortega R, Guzmán C, Alvarez J (2015). Molecular characterization of several *Wx* alleles in durum wheat. Biol. Plant.

[21] Divashuk M, Klimushina M, Karlov G (2011). Molecular genetic characteristics of the *Wx-B1e* allele from common wheat and applicability of the DNA markers for its identification. Russ. J. Genet..

[22] Guzmán C, Caballero L, Álvarez JB (2011). Molecular characterisation of the *Wx-B1* allelic variants identified in cultivated emmer wheat and comparison with those of durum wheat. Mol. Breed..

[23] Ayala M (2015). Molecular characterization of waxy alleles in three subspecies of hexaploid wheat and identification of two novel *Wx-B1* alleles. Theor. Appl. Genet..

[24] Yamamori M (2009). Amylose content and starch properties generated by five variant *Wx* alleles for granule-bound starch synthase in common wheat (*Triticum aestivum* L.). Euphytica.

[25] Yamamori M, Yamamoto K (2011). Effects of two novel *Wx-A1* alleles of common wheat (*Triticum aestivum* L.) on amylose and starch properties. J. Cereal Sci..

[26] Zhang LL (2017). Transposon insertion resulted in the silencing of *Wx-B1n* in Chinese wheat landraces. Theor. Appl. Genet..

[27] Guzmán C (2015). Molecular characterization of two novel null waxy alleles in Mexican bread wheat landraces. J. Cereal Sci..

[28] Rodriguez-Quijano M, Nieto-Taladriz MT, Carrillo JM (1998). Polymorphism of waxy proteins in Iberian hexaploid wheats. Plant Breed..

[29] Luo M (2019). A single-base change at a splice site in *Wx-A1* caused incorrect RNA splicing and gene inactivation in a wheat EMS mutant line. Theor. Appl. Genet..

[30] Graybosch R (1998). Identification and characterization of US wheats carrying null alleles at the *wx* loci. Cereal Chem..

[31] Kiribuchi-Otobe C (1998). Wheat mutant with waxy starch showing stable hot paste viscosity. Cereal Chem..

[32] Yasui T (2006). Waxy and low-amylose mutants of bread wheat (*Triticum aestivum* L.) and their starch, flour and grain properties. JARQ.

[33] Yasui T, Ashida K, Sasaki T (2009). Chain-length distribution profiles of amylopectin isolated from endosperm starch of waxy and low-amylose bread wheat (*Triticum aestivum* L.) lines with common genetic background. Starch-Stärke.

[34] Yanagisawa T, Kiribuchi-Otobe C, Yoshida H (2001). An alanine to threonine change in the Wx-D1 protein reduces GBSS I activity in waxy mutant wheat. Euphytica.

[35] Slade AJ (2005). A reverse genetic, nontransgenic approach to wheat crop improvement by TILLING. Nat. Biotechnol..

[36] Botticella E (2018). Combining mutations at genes encoding key enzymes involved in starch synthesis affects the amylose content, carbohydrate allocation and hardness in the wheat grain. Plant Biotechnol. J..

[37] Momma M, Fujimoto Z (2012). Interdomain disulfide bridge in the rice granule bound starch synthase I catalytic domain as elucidated by X-ray structure analysis. Biosci. Biotechnol. Biochem..

[38] Wang BB, Brendel V (2006). Genomewide comparative analysis of alternative splicing in plants. Proc. Natl. Acad. Sci..

[39] Barbazuk WB, Fu Y, McGinnis KM (2008). Genome-wide analyses of alternative splicing in plants: Opportunities and challenges. Genome Res..

[40] Huang X (2008). Genome-wide analysis of transposon insertion polymorphisms reveals intraspecific variation in cultivated rice. Plant Physiol..

[41] Warnefors M, Pereira V, Eyre-Walker A (2010). Transposable elements: Insertion pattern and impact on gene expression evolution in hominids. Mol. Biol. Evol..

[42] Wilkinson M, Lenton J, Holdsworth M (2005). Transcripts of *Vp-1* homoeologues are alternatively spliced within the Triticeae tribe. Euphytica.

[43] Yang Y (2007). Development and validation of a *Viviparous-1* STS marker for pre-harvest sprouting tolerance in Chinese wheats. Theor. Appl. Genet..

[44] Vrinten P, Nakamura T, Yamamori M (1999). Molecular characterization of *waxy* mutations in wheat. Mol. Gen. Genet..

[45] Van Hung P, Maeda T, Morita N (2006). Waxy and high-amylose wheat starches and flours—characteristics, functionality and application. Trends Food Sci. Technol..

[46] Monari AM (2005). Molecular characterization of new waxy mutants identified in bread and durum wheat. Theor. Appl. Genet..

[47] Yamamori M, Yasui T (2016). Combination of null, variant, and mutant *Wx* alleles in common wheat leads to amylose variations ranging from waxy to normal. Crop Sci..

[48] Dong C (2009). A modified TILLING method for wheat breeding. Plant Genome.

[49] Sestili F (2010). Production of novel allelic variation for genes involved in starch biosynthesis through mutagenesis. Mol. Breed..

[50] Chen GX (2016). Biosynthesis and regulation of wheat amylose and amylopectin from proteomic and phosphoproteomic characterization of granule-binding proteins. Sci. Rep..

[51] Yamamori M, Endo T (1996). Variation of starch granule proteins and chromosome mapping of their coding genes in common wheat. Theor. Appl. Genet..

