# Supplementary material for: Identification and molecular characterization of mutant line deficiency in three waxy proteins of common wheat (Triticum aestivum L.)
Source: Sci Rep. 2021 Feb 10;11:3510. doi: 10.1038/s41598-021-82865-2 (PMC7876011; doi:10.1038/s41598-021-82865-2)
Supplement: Supplementary file 1 — Supplementary Information. [file 41598_2021_82865_MOESM1_ESM.pdf]

Supplementary information for the paper

"Identification and molecular characterization of mutant line deficiency in three waxy proteins of common wheat (*Triticum aestivum* L.)"

Qian Liu[1 †], Yaping Hu[2, 1, 3 †], Mengyun Hu[1], Lijing Sun[1], Xiyong Chen[1], Qianying Li[1], Peinan Wang[1], Li-an Wang[3], Yingjun Zhang[1 *], Hui Li[1 *]

[1] Institute of Cereal and Oil Crops, Hebei Academy of Agriculture and Forestry Sciences, Hebei Laboratory of Crop Genetics and Breeding, 162 Hengshan Street, Shijiazhuang 050035, China

[2] Xingtai Medical College, 618 Gangtie North Road, Xingtai 054000, China

[3] College of Life Sciences, Hebei Normal University, 20 Road East. 2nd Ring South, Shijiazhuang 050024, China

Qian Liu, e-mail: liuqian_mbb@126.com

Yaping Hu, e-mail: huyaping1983@126.com

Mengyun Hu, e-mail: ziren80@163.com

Lijing Sun, e-mail: sunlijing2010@163.com

Xiyong Chen, e-mail: chenxiyong369@126.com

Qianying Li, e-mail: 415025147@qq.com

Peinan Wang, e-mail: 2455258978@qq.com

Li-an Wang, e-mail: wlian1965@126.com

[†]Qian Liu and Yaping Hu contributed equally to this work.

[*]Author for correspondence:

Yingjun Zhang, e-mail: zhangyingjun1977@163.com

Hui Li, e-mail: zwslihui@163.com

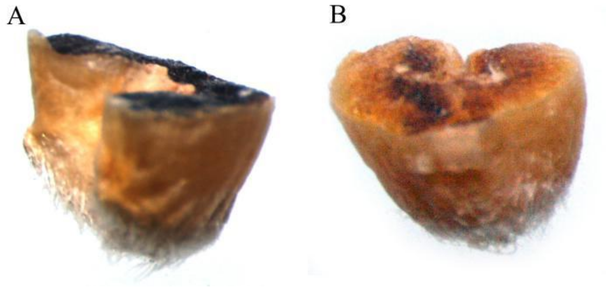

Fig. S1. Identification of waxy protein-deficient mutant line stained with I_2-KI solution. **A:** wild-type; **B:** Wx-null line.

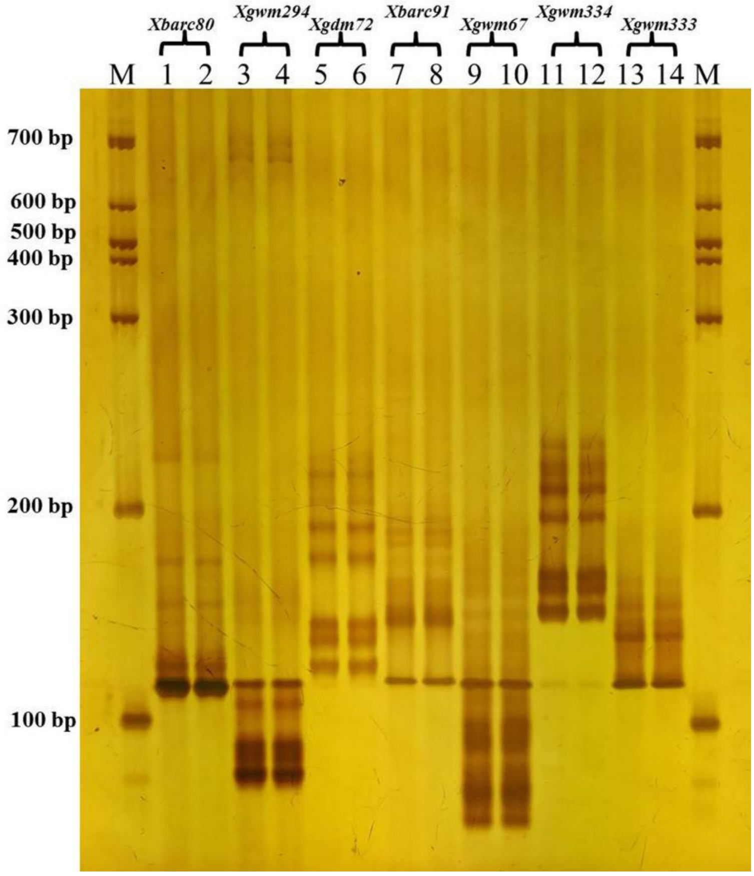

Fig. S2. SSR analysis of the wild-type and Wx-null line. **1, 3, 5, 7, 9, 11, 13,** wild-type; **2, 4, 6, 8, 8, 10, 12, 14,** Wx-null line; **M,** DNA marker. The names of SSR markers are shown above the figure.

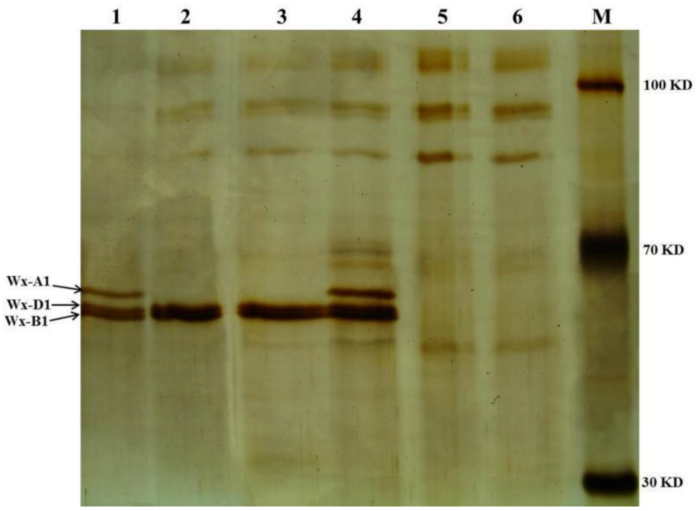

Fig. S3. SDS-PAGE analysis of the waxy protein mutant line. **1,** wild-type; **2, 3,** Wx-A null line; **4,** wild-type; **5, 6,** Wx-null line; **M,** protein molecular marker.

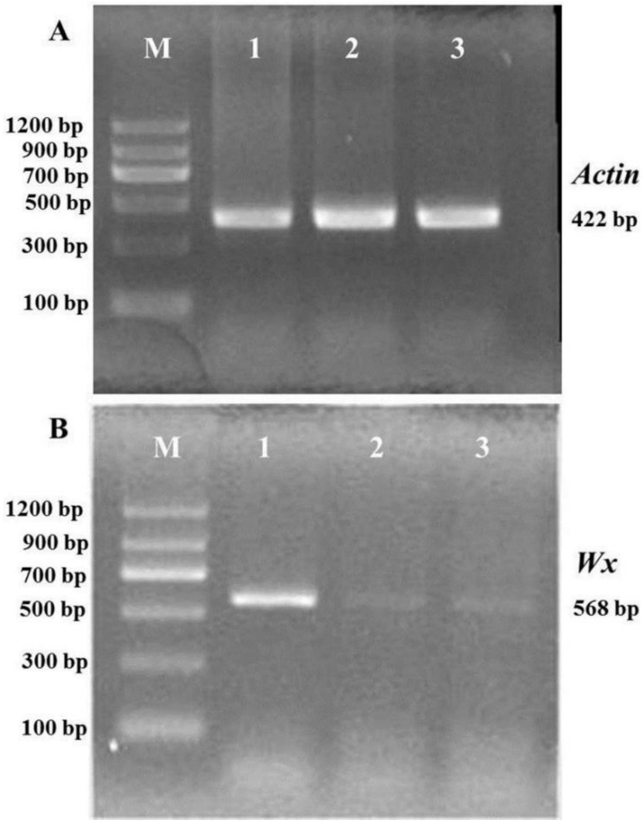

Fig. S4. Expression analysis of *Wx* gene using RT-PCR. Gene names shown on the right side. **A:** *Actin* gene; **B:** *Wx* gene. **M**, DNA marker; **1**, wild-type; **2, 3,** Wx-null line.

Table S1 Primer sets used in this study

Primer set	Sequences (5'-3')	Tm (^{o}C)	Amplified target
A1	F: ATGGCGGCTCTGGTCACGTC<br>R: CGCAAAGACTCACTGAAATTATC	57	*Wx-A1* gene
A2	F: ACCCGCATGGTGTTTGATAATTTCAGTG<br>R: AGAATGCCACCTAGCCATGAAATGGAGT	62	
A3	F: CGCTCTGCATATCAATTTTGCGGTTC<br>R: CCTGCAATGCATTCGATCAGTCAGTC	62	
A4	F: GTTCTCCACCAACGATCGACCGACATT<br>R: ATCGGCCCTTCACTCTTAGTTGTTCCAG	63	
A5	F: CGGTAAACGCATCCTCCTTCAGT<br>R: CTTCAGGGAGCGGCGACGTTC	60	
B1	F: ATGGCGGCTCTGGTCACGTC<br>R: GACACTCTAGTAGCTGCTGGGT	60	*Wx-B1* gene
B2	F: GGTAAGATCAACAACACCCAGCAGCTA<br>R: AACCAGCAATCACCGGAAGAAATCTTTG	61	
B3	F: CCACCCACACACCCACACAAAGAT<br>R: TTTACACAAGGGATCGACGAGCCTAC	62	
B4	F: GATTCCTTATCTCCCCGCGTATC<br>R: CTTCAGGGAGCGGCGACGTTC	60	
D1	F: ATGGCGGCTCTGGTCACGTC<br>R: GATTACACACACTCTAGCTTGAAC	57	*Wx-D1* gene
D2	F: TCGTCGTCTCAACCTTGATAGGCATGGTGAT<br>R: GAACCGCAAAATTGATATGCCTGTTTCA	60	
D3	F: TGAAACAGGCATATCAATTTTGCGGTTC<br>R: TCGATCATTCCTTAGGTCTGCTTGATCG	60	
D4	F: CATCATCGAGCCCGCAACCCGAC<br>R: CTTCAGGGAGCGGCGACGTTC	62	
Actin	F: GTTCCAATCTATGAGGGATACACGC<br>R: GAACCTCCACTGAGAACAACATTACC	59	*Actin* gene
Wx-RT	Wx-F：CTGGAGAAGGTCCGGGGCA	59	RT-PCR

	Wx-R：ACTCGCTGACGTCCATGCCG		primers for *Wx* gene

Legends:

Table S1 Primer sets used in this study.

Fig. S1 Identification of waxy protein-deficient mutant line stained with I_2-KI solution. **A:** wild-type; **B:** Wx-null line.

Fig. S2. SSR analysis of the wild-type and Wx-null line. **1, 3, 5, 7, 9, 11, 13,** wild-type; **2, 4, 6, 8, 8, 10, 12, 14,** Wx-null line; **M,** DNA marker. The names of SSR markers are shown above the figure.

Fig. S3 SDS-PAGE analysis of the waxy protein mutant line. **1,** wild-type; **2, 3,** Wx-A null line; **4,** wild-type; **5, 6,** Wx-null line; **M,** protein molecular marker.

Fig. S4 Expression analysis of *Wx* gene using RT-PCR. Gene names shown on the right side. **A:** *Actin* gene; **B:** *Wx* gene. **M**, DNA marker; **1**, wild-type; **2, 3,** Wx-null line.
